# Gene4MND: An Integrative Genetic Database and Analytic Platform for Motor Neuron Disease

**DOI:** 10.3389/fnmol.2021.644202

**Published:** 2021-04-01

**Authors:** Guihu Zhao, Zhen Liu, Mengli Wang, Yanchun Yuan, Jie Ni, Wanzhen Li, Ling Huang, Yiting Hu, Pan Liu, Xiaorong Hou, Jifeng Guo, Hong Jiang, Lu Shen, Beisha Tang, Jinchen Li, Junling Wang

**Affiliations:** ^1^Department of Neurology, Xiangya Hospital, Central South University, Changsha, China; ^2^Department of Geriatrics, Xiangya Hospital, Central South University, Changsha, China; ^3^National Clinical Research Center for Geriatric Diseases, Xiangya Hospital, Central South University, Changsha, China; ^4^Department of Neurology, The Third Xiangya Hospital, Central South University, Changsha, China; ^5^Hunan Key Laboratory of Medical Genetics, Center for Medical Genetics, School of Life Sciences, Central South University, Changsha, China; ^6^Key Laboratory of Hunan Province in Neurodegenerative Disorders, Central South University, Changsha, China

**Keywords:** amyotrophic lateral sclerosis, motor neuron disease, one-stop online database, gene ontology analysis, immunomodulation, gene

## Introduction

Motor neuron diseases (MND) refer to a wide and heterogeneous expanding group of neurodegenerative disorders involving the upper and lower motor neurons, which are characterized by rapidly progressive weakness leading to paralysis and eventually death from respiratory failure. Clinically, it is mainly represented by amyotrophic lateral sclerosis (ALS), progressive muscular atrophy (PMA), primary lateral sclerosis (PLS), and progressive bulbar palsy (PBP). As the most common type of MND, the number of patients with ALS is rapidly increasing in part because of population aging. It was estimated that 330,918 patients had been diagnosed with ALS worldwide in 2016 (Marin et al., 2017), but significant geographical differences exist. To date, the pathologic mechanisms of MND are still unclear, and effective treatments are not available yet. Thus, further genetic and molecular research on the underlying mechanisms is warranted.

In recent years, significant progress has been made to dissect the genetic architecture of MND. Promising MND candidate genes and rare variants (Rosen, 1993; Gitcho et al., 2008; Sreedharan et al., 2008; Zou et al., 2017) have been revealed by genetic linkage and association, gene expression studies, and convergent functional genomics. Multiple highly significant risk variants have also been successfully identified in genome-wide association studies (GWAS) (Nicolas et al., 2018; Farhan et al., 2019). In addition, differentially expressed genes (DEGs) (Wang et al., 2006; Lederer et al., 2007; Shtilbans et al., 2011; Gregory et al., 2020) and differentially methylated genes (DMGs) (Martin and Wong, 2013; Belzil et al., 2014; Ebbert et al., 2017; Coppedè et al., 2018) have also been associated with MND pathogenesis. Despite these great advances in the genetics and genomics of MND, it is difficult to retrieve pertinent information and to annotate it in an efficient manner. Therefore, it is necessary to conduct thorough collection, systematic integration and detailed annotation of existing genes and mutations underlying MND.

In this study, we developed a one-stop database of MND-related genes and variants, Gene4MND, to facilitate analysis of genes and variants related to MND. By systematically searching and manually reviewing the literature in PubMed, we included most of the genes and variants related to MND. Combined with popular genomic data sources, various functional annotation data were integrated into the database. Furthermore, we adopted a scoring system based on the genetic evidence to prioritize genes and performed functional analysis of the prioritized genes.

## Methods

### Data Collection

To obtain a complete list of genes and variants relevant to MND, systematic searches were performed in the PubMed database prior to August 30, 2020, and then, MND-related publications were reviewed manually. Studies were included if they met all of the following criteria: (1) studies with genetic information about rare variants and relatively common variants, copy number variants (CNVs), DEGs and DMGs in MND patients; (2) the diagnosis criteria were described in detail; and (3) the sequencing method and loci of variants were provided. In addition, information about DEGs and DMGs were also collected from an MND animal model. Reviews, expert opinions, and editorials were excluded. Consequently, a total of 478 studies met the inclusion criteria for further information extraction. Genetic information, basic information and clinical data were extracted through in-depth reading of the full text of each publication. Of note, GGGGCC hexanucleotide repeat expansions in the non-coding region of *C9orf72* and *CAG* trinucleotide repeat expansions in the *ATXN2* gene, which have been extensively researched (Vance et al., 2006; DeJesus-Hernandez et al., 2011; Renton et al., 2011; Majounie et al., 2012; Jiao et al., 2014), were considered common genetic causes for ALS. Thus, the nucleotide repeat variations of those two genes were cataloged independently. A summary of major data types is shown in Supplementary Table 1.

### Functional Annotation

Sixty-three genomic data sources were integrated to annotate each variant and gene (Supplementary Table 2). At the variant level, ANNOVAR (Wang et al., 2010) was used for comprehensive annotation. It produced not only functional effects of variants (frameshift, non-frameshift, synonymous, non-synonymous, stopgain, stoploss, splicing, and non-coding) but also disease- and phenotype-related information for functional implications. In addition, we annotated the functional consequences of variants through 23 *in silico* predictive algorithms and provided allele frequencies in different populations of public databases.

At the gene level, a comprehensive annotation for each gene was provided, including basic gene information, gene function (molecular function (MF), gene ontology (GO), protein-protein interaction (PPI), and pathway), disease- and phenotype-related information, gene expression patterns in the human brain, variants in different populations, and drug-gene interactions, as in our previous databases, VarCards and Gene4Denovo (Li et al., 2018; Zhao et al., 2020).

### Gene Prioritization

To identify high-confidence genes by their relevance to MND, gene prioritization (except for *C9orf72* and *ATXN2*) was conducted by combining the different types of genetic evidence as follows: a score of five was assigned to CNVs and rare loss-of-function (LoF) variants including stopgain, stoploss, splicing sites, single-nucleotide variants (SNVs), and frameshift indels; scores of 3, 2, and 1 were assigned to rare deleterious missense variants, rare non-synonymous SNVs, and non-synonymous SNVs, respectively; a score of 1–3 was assigned to single-nucleotide polymorphisms (SNPs), DEGs, and DMGs based on their *p*-values (Supplementary Table 3). Rarity was defined as a minor allele frequency <0.0001 based on gnomAD.

### Functional Analysis Based on the Prioritized Genes

Further data mining based on the prioritized genes was performed to schematize the functional relevance of MND-related genes, and genes with a total score of at least 4 were considered prioritized genes. GO analysis of the biological process (BP), cellular component (CC), and MF levels and Kyoto Encyclopedia of Genes and Genomes (KEGG) pathway analysis were performed using the cluster Profiler package. An adjusted *p*-value < 0.01 was considered statistically significant, and the visualization of results was performed with the GO plot package.

A PPI network was constructed using the STRING database, and the minimum required interaction score was 0.4. The core cluster was extracted using the MCODE algorithm in Cytoscape software with a node score cut-off of 0.2 and K-core of 4. GO analysis at the BP, CC, and MF levels and KEGG analysis were further performed based on the genes in the core clusters.

## Results

### Data and Database Interface

**Figure 1:**
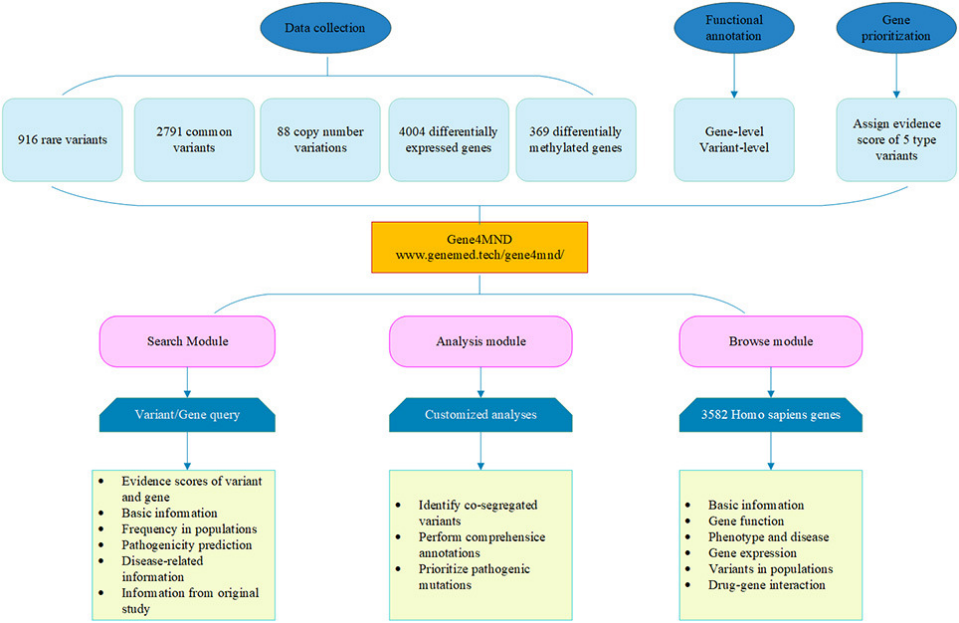
Overview of the Gene4MND database. Multiple types of data from various sources were collected and curated. Systematic data and functional annotation from more 60 genomic data sources were integrated into Gene4MND, which contains three core modules providing comprehensive description information and customized analysis.

Consequently, except for the polynucleotide expansion of *C9orf72* and *ATXN2*, 916 rare variants, 2,791 common variants, 88 CNVs, 4,004 DEGs, 369 DMGs, and the corresponding detailed genetic and clinical information were integrated into the Gene4MND database (Figure 1, Supplementary Table 1).

A user-friendly web interface, Gene4MND (www.genemed.tech/gene4mnd/), was developed, supported by versatile browsing and searching functionalities, as in our previous databases and web servers (Li et al., 2018; Zhao et al., 2020). All of the data were stored in a MySQL database. Users can access the genetic data or extended analysis results freely through this web interface.

The web interface of Gene4MND contains three core modules: the Search, Analysis, and Browse modules (Figure 1).

(1) **Search module**. This module provides two options, Quick Search and Advanced Search, to query variants and genes. Quick Search supports several common search terms, such as gene symbol, genomic region, and variant. In addition to those terms, Advanced Search supports more terms, such as cytoband, variant, transcript, and genomic coordinate, and it supports simultaneously searching several terms. For each query, Gene4MND will generate a summary report that summarizes the evidence score of the gene from the variant types of the collected genetic data, including the rare variant score, associated SNP score, CNV score, DEG score, DMG score, and combined score. The detailed information of the five types of variants is provided in the subsequent five tables. Of note, in the rare variants section, the link Expand row will provide the *in silico* missense prediction, allele frequency in the population, disease information, and information from the original study of rare variants.
(2) **Analysis module**. Users can perform customized analyses with this module. After inputting genetic data (VCF4 format) and choosing the genotype information of samples, the genetic variants will be analyzed by Gene4MND using default parameters. Of note, users can also specify their cut-off values for quality control, annotation data sources, and parameters for identifying rare damaging variants. To perform comprehensive annotations, the databases for basic information annotation, pathogenicity prediction of missense variants, allele frequency in the variant population, and clinical-related data need to be specified. Gene4MND will send a link to the user by e-mail to download the results when the analysis is complete.
(3) **Browse module**. A total of 3,817 *Homo sapiens* genes were collated and integrated into Gene4MND. By sourcing from more than 60 genomic data sources, a one-stop interface of a given gene is provided, including basic information, such as the primary information of genes, gene function, phenotype and disease data; gene expression, which provides the spatiotemporal expression profiles and expression in different tissues of specific genes; variants in different populations; and drug-gene interactions.
(4) **Upload and download module**. In addition, this database also provides the ability to upload and download modules, which encourages all laboratories and researchers to upload their variant files and download the datasets that they need.

### Functional Analysis Based on the Prioritized Genes

**Figure 2:**
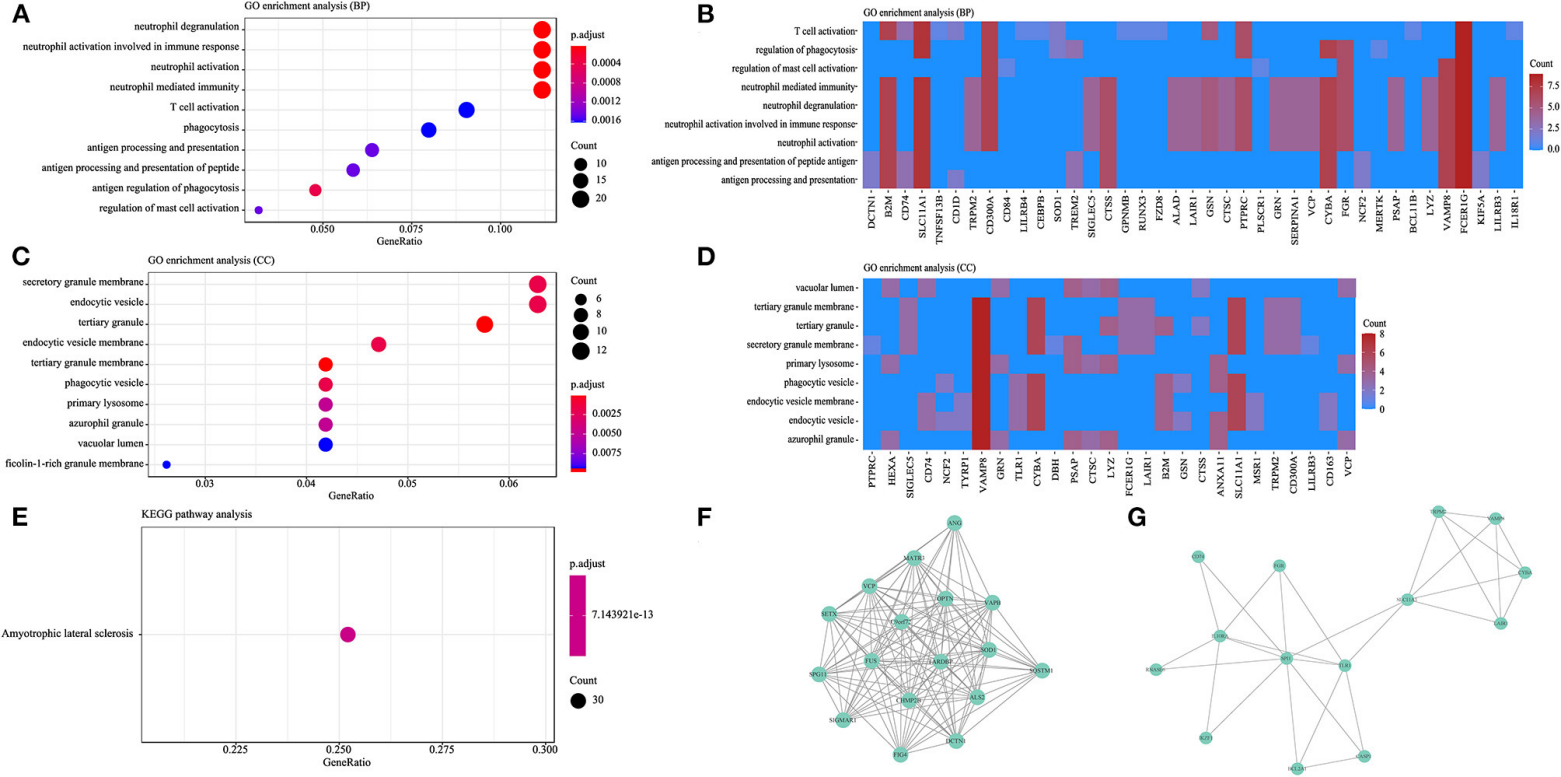
Functional analysis based on the prioritized genes. **(A)** GO analysis showing the top 10 GO terms related to BP. **(B)** Heatmap showing the BP clustering of the prioritized genes. **(C)** GO analysis showing the top 10 GO terms related to CC. **(D)** Heatmap showing the CC clustering of the prioritized genes. **(E)** KEGG pathway analysis. **(F)** Core cluster 1 extracted from the PPI network. **(G)** Core cluster 2 extracted from the PPI network.

A total of 200 genes with an evidence score >4 were included as prioritized genes, and the top 10 prioritized genes are as follows: *SOD1, FUS, TARDBP, TBK1, OPTN, VCP, SETX, FIG4, SQSTM1*, and *VAPB*. Considering that nucleotide repeat expansions in the *C9orf72* and *ATXN2* genes are commonly considered genetic causes of MND, the *C9orf72* and *ATXN2* genes were also included in the functional analysis. GO analysis indicated that prioritized genes were significantly enriched in 51 GO terms, including 41 GO terms (80.3%) related to BP and 10 GO terms (19.7%) related to CC, while no GO terms were related to MF. Among the GO terms related to BP, most prioritized genes were included in pathways were related to the immune response (Figures 2A,B), such as neutrophil activation and degranulation, T cell activation, regulation of cytokine production, phagocytosis, and antigen processing and presentation. Among the GO terms related to CC, the majority of prioritized genes were involved in cell granule formation, secretion, and phagocytosis (Figures 2C,D). KEGG analysis revealed that prioritized genes were enriched in the ALS pathway (Figure 2E).

Based on the prioritized genes, we extracted two clusters from the constructed PPI network (Figures 2F,G). Genes in cluster 1 were mainly involved in endomembrane system organization, endosomal transport, macro-autophagy, the neuronal cell body, vesicle organization, and the growth cone (Supplementary Table 4). Genes in cluster 2 were mainly involved in the following pathways: positive regulation of cytokine production, neutrophil degranulation, neutrophil activation involved in the immune response, neutrophil activation, and neutrophil-mediated immunity (Supplementary Table 4).

## Discussion

With the development of sequencing technologies, a growing number of genetic insights into MND have been revealed (Brenner and Weishaupt, 2019; Mathis et al., 2019; Mejzini et al., 2019). Nevertheless, the pathophysiology of MND remains largely unknown, and the scattered results create a major challenge for interpreting close relationships between genotypes and phenotypes. Therefore, we constructed a comprehensive genetic resource for MND, Gene4MND, to systematically integrate multiple types of data from various studies and to obtain meaningful biological information from genetic findings of MND.

Despite the advances of other available databases, such as ALSdb (http://alsod.org/), ALSoD (https://alsod.ac.uk/output/gene.php) (Yoshida et al., 2010), and Project MinE (https://www.als.org/) (Consortium, 2018), Gene4MND, which is designed as a one-stop database of MND variants and genes, has several advantages. First, the web interface of Gene4MND is more user-friendly, and it provides a more intuitive online interface for researchers without sufficient bioinformatics skills to access the first-hand genetic, genomic and clinical information of MND-related variants within a short time. Second, the Gene4MND database not only integrates all of the collected genetic information but also retrieves information from more than 60 genomic data sources. Third, the Gene4MND database evaluates and scores each piece of evidence for specific variants and integrates scattered genetic, genomic, and clinical data sources to prioritize disease-causing or disease risk variations. Of note, Gene4MND provides a platform for custom analysis, and users will be able to flexibly prioritize candidate variations and genes based on genetic data and different criteria according to the needs of the study. Finally, as a database of MND, Gene4MND provides a repository for researchers to further analyse the existing evidence to understand the pathogenesis of ALS. Based on the prioritized genes, GO, KEGG and PPI analysis can be used to help the user understand the complex mechanisms underlying ALS. For example, using GO analysis, we revealed that most MND-related genes are involved in the immune response, which is consistent with previous studies (Mantovani et al., 2009; Hooten et al., 2015; Brown and Al-Chalabi, 2017), suggesting that immunomodulation plays important roles in the pathogenesis of MND.

Certainly, there are several issues that should be emphasized. On the one hand, the evidence of clinically significant variations from different studies with differences in criteria and methods of assessing the pathogenicity of genetic variants may lead to bias. On the other hand, users should note the potential limitations with regard to the specificity and sensitivity of the *in silico* prediction tools. Notably, a continuous rapid increase in data generation and improvement of data quality in MND research in the future is expected, and thus, we will routinely update the database.

In conclusion, Gene4MND is a dedicated repository, platform and communication warehouse for MND that aims to accelerate genetic consulting, enhance the understanding of the diseases and facilitate MND research.

## Data Availability Statement

The datasets presented in this study can be found in online repositories. The names of the repository/repositories and accession number(s) can be found in the article/Supplementary Material.

## Author Contributions

JL, JW, JG, HJ, LS, and BT: database design. ZL, GZ, YY, JN, YH, JW, WL, PL, and XH: data collection. GZ, ZL, JW, and JL: database construction and maintenance. ZL, GZ, and MW: data analysis. MW, LH, ZL, GZ, JL, and JW: manuscript writing. All authors contributed to the article and approved the submitted version.

## Conflict of Interest

The authors declare that the research was conducted in the absence of any commercial or financial relationships that could be construed as a potential conflict of interest.
