# Supplementary material for: Gene4MND: An Integrative Genetic Database and Analytic Platform for Motor Neuron Disease
Source: Front Mol Neurosci. 2021 Apr 1;14:644202. doi: 10.3389/fnmol.2021.644202 (PMC8047132; doi:10.3389/fnmol.2021.644202)
Supplement: Supplementary file 1 [file Table_1.DOC]

Table S1. Summary of the major data types

| Data type | publications reviewed | variants | genes |
| --- | --- | --- | --- |
| rare variants | 391 | 916 | 206 |
| C9orf72 repeat | 16* | 1 | 1 |
| common variants | 22 | 2791 | >2000(2179) |
| copy number variants | 14 | 88 regions | >100 |
| differentially expressed | 24 |  | >4000(4004) |
| differentially methylated | 11 |  | 369 |

Table S2. Summary of the integrated data sources in Gene4MND

| Category | Data source |
| --- | --- |
| Allele frequency | GnomAD, ExAC, 1000Genomes, ESP6500, Kaviar, HRC |
| Missense prediction | CADD, CDTS, CScape, DANN, Eigen, FATHMM-MKL, FATHMM-XF, FIRE, fitCons2, FunSeq2, GenoCanyon, LINSIGHT, ReMM, Divan, Orion, PAFA, DVAR, regBase, ncER |
| Disease-related | InterVar, COSMIC70, ICGC, nci60, dbSNP, interPro, ClinVar, OMIM, denovo-db, MGI, HPO |
| Basic information | Entrez, MIM, HGNC, Ensembl, GeneCards, RVIS, LoFtool, heptanucleotide context intolerance score, GDI, Episcore, pLI score, NCBI Gene |
| Gene function | UniProtKB, GO, InBio Map, NCBI BioSystems |
| Gene expression | GTEx, The Human Protein Atlas |
| Homology | HomoloGene, TreeFam |
| Target drug | DGIdb |

gnomAD, Genome Aggregation Database; ExAC, Exome Aggregation Consortium; 1000Genomes, 1000 Genomes Project; ESP, Exome Sequencing Project; HRC, Haplotype Reference Consortium; CADD, combined annotation dependent depletion; CDTS, context-dependent tolerance score; fitCons2, fitness consequences of functional annotation; ReMM, regulatory Mendelian mutation; Divan, disease-specific variant annotation; ncER, non-coding essential regulation; ICGC, International Cancer Genome Consortium; dbSNP, Single Nucleotide Polymorphism Database; OMIM, online Mendelian inheritance in man; HPO,  human phenotype ontology; RVIS, residual variation intolerance score; GDI, gene damage index;  UniProtKB,  UniProt Knowledgebase; GO, gene ontology; GTEx, genotype-tissue expression; TreeFam, Tree Families Database; DGIdb, Drug-Gene Interaction Database

**Table S3. Weight scheme of various genetic data for prioritizing candidate genes by p value.**

| Genetic data | Score = 5 | Score = 3 | Score = 2 | Score = 1 |
| --- | --- | --- | --- | --- |
| Associated SNPs | - | P≤10E-12 | 10E-12<P≤10E-8 | 10E-8<P≤10E-4 |
| DEGs | - | P≤10E-6 | 10E-6<P≤10E-4 | 10E-4<P≤10E-2 |
| DMGs | - | P≤10E-6 | 10E-6<P≤10E-4 | 10E-4<P≤10E-2 |

DEGs, differential expression; DMGs, differential DNA methylation.

Table S4. Top 5 GO terms and KEGG pathways based on genes in cluster 1 and 2.

| Cluster | Ontologies | Description | Gene_Count | p.adjust |
| --- | --- | --- | --- | --- |
| Cluster 1 | GO_BP | endomembrane system organization | 8 | 1.35E-04 |
|  | GO_BP | endosomal transport | 5 | 1.35E-04 |
|  | GO_BP | macroautophagy | 5 | 1.35E-04 |
|  | GO_BP | cellular response to unfolded protein | 4 | 1.35E-04 |
|  | GO_BP | vesicle organization | 5 | 1.35E-04 |
|  | GO_CC | growth cone | 5 | 1.73E-05 |
|  | GO_CC | site of polarized growth | 5 | 1.73E-05 |
|  | GO_CC | neuronal cell body | 6 | 9.24E-05 |
|  | GO_CC | distal axon | 5 | 9.32E-05 |
|  | GO_CC | cytoplasmic stress granule | 3 | 5.26E-04 |
|  | GO_MF | polyubiquitin modification-dependent protein binding | 3 | 1.42E-03 |
|  | GO_MF | K63-linked polyubiquitin modification-dependent protein binding | 2 | 8.45E-03 |
|  | GO_MF | modification-dependent protein binding | 3 | 8.45E-03 |
|  | GO_MF | ionotropic glutamate receptor binding | 2 | 8.45E-03 |
|  | GO_MF | Rab GTPase binding | 3 | 8.45E-03 |
|  | KEGG | amyotrophic lateral sclerosis | 17 | 2.10E-22 |
| Cluster 2 | GO_BP | positive regulation of cytokine production | 6 | 1.35E-04 |
|  | GO_BP | neutrophil degranulation | 6 | 1.35E-04 |
|  | GO_BP | neutrophil activation involved in immune response | 6 | 1.35E-04 |
|  | GO_BP | neutrophil activation | 6 | 1.35E-04 |
|  | GO_BP | neutrophil mediated immunity | 6 | 1.35E-04 |
|  | GO_CC | tertiary granule membrane | 5 | 1.17E-07 |
|  | GO_CC | tertiary granule | 5 | 2.55E-06 |
|  | GO_CC | endocytic vesicle membrane | 5 | 2.55E-06 |
|  | GO_CC | phagocytic vesicle membrane | 4 | 4.90E-06 |
|  | GO_CC | specific granule membrane | 4 | 8.12E-06 |
